# Normativity, interpretation, and Bayesian models

**DOI:** 10.3389/fpsyg.2014.00332

**Published:** 2014-05-15

**Authors:** Mike Oaksford

**Affiliations:** Department of Psychological Sciences, Birkbeck College, University of LondonLondon, UK

**Keywords:** psychology of reasoning, Bayesian models, Bayesian argumentation, radical interpretation, Donald Davidson, evaluative normativity

## Abstract

It has been suggested that evaluative normativity should be expunged from the psychology of reasoning. A broadly Davidsonian response to these arguments is presented. It is suggested that two distinctions, between different types of rationality, are more permeable than this argument requires and that the fundamental objection is to selecting theories that make the most rational sense of the data. It is argued that this is inevitable consequence of radical interpretation where understanding others requires assuming they share our own norms of reasoning. This requires evaluative normativity and it is shown that when asked to evaluate others’ arguments participants conform to rational Bayesian norms. It is suggested that logic and probability are not in competition and that the variety of norms is more limited than the arguments against evaluative normativity suppose. Moreover, the universality of belief ascription suggests that many of our norms are universal and hence evaluative. It is concluded that the union of evaluative normativity and descriptive psychology implicit in Davidson and apparent in the psychology of reasoning is a good thing.

Elqayam and Evans (2011) have argued against *evaluative* normativity having any role in psychological theories of reasoning. They contrast evaluative normativity with *directive* normativity. They argue that directive normativity is conditional and perfectly consistent with programs in cognitive science like rational analysis (Anderson, 1990; Oaksford and Chater, 1998, 2007). Consequently, they have no problem with formulations like, *if you want to be well adapted to your environment then you should act in a Bayes optimum fashion in classification, decision and prediction*. However, what we can’t apparently assert is the unconditional *you should act in a Bayes optimum fashion in classification, decision, and prediction*. This is an evaluative claim suggesting in some absolute sense that this is the right way to behave. In particular, they observe that if there were an alternative normative theory of what constitutes being well adapted to your environment, citing empirical evidence to distinguish between these two normative theories would commit the *is-ought* fallacy. Consequently, evaluative normativity should be expunged from psychological theorizing about reasoning.

In this paper, I pursue a broadly Davidsonian (Davidson, 2004) response to Elqayam and Evans’ (2011). In the first section, *Types of Rationality*, I set up the argument by observing that two distinctions they make, between *instrumental* and *normative* rationality and between *directive* and *evaluative* rationality, are far more permeable than they require. I conclude that Elqayam and Evans (2011) primary objection is to the suggestion that we should pick the theory that makes the most rational sense of our data. In the second section, *Interpretation, Argumentation, and Rationality*, I argue that this is inevitable consequence of Davidson’s account of radical interpretation. On Davidson’s view, rationality is a social construct where to interpret others’ statements requires that we adopt a principle of charity, i.e., they share the same norms as ourselves. Davidson’s account suggests attributing people with intentional states like beliefs requires evaluative normativity. I then show that in the social context of argumentation, a third person argument evaluation methodology yields close conformity to rational Bayesian norms. Participants are quite capable of evaluating others arguments. I conclude that this ubiquitous human behavior is something that psychology must explain. In the final section, *How Many Rational Norms Are There*? I argue that logic and probability theory are not really competing norms, the important psychological question is whether beliefs are binary or graded. Moreover, following Davidson, I question Elqayam and Evans (2011) grounds for normative relativism. In conclusion, I suggest that while there are many outstanding problems and exceptions, the continuing union of evaluative normativity and descriptive psychology apparent in the psychology of reasoning is a good thing.

## TYPES OF RATIONALITY

Stanovich (2011) argued that Elqayam and Evans (2011) drive a wedge between Bayesian probability theory, which they regard as an account of normative rationality, and *instrumental* rationality. Instrumental or practical rationality, which Elqayam and Evans (2011) endorse, provides a suitable means for achieving one’s goals regardless of the nature of those goals. However, as Stanovich (2011) observes, this is a difficult wedge to drive home given that the standard justification for the laws of subjective probability are given by the Dutch book theorem (Vineberg, 2011). For each of the laws of probability theory, this theorem establishes that violating them would leave an agent open to making bets they cannot win. The *converse* Dutch book theorem then establishes that these laws are instrumentally rational because conforming to them prevents taking self-defeating actions. This instrumentally rational justification can then be provided with a directively rational formulation: *if an agent wishes to avoid making bets they cannot win, then they should conform to the laws of probability theory*. This conditional formulation just restates the *converse* Dutch book theorem. So this formulation involves making conformity to the normative theory conditional on that normative theory’s rational justification. The justification for probability theory is instrumental [other epistemic justifications, based on maximizing accuracy, are equally instrumental (Joyce, 1998)]. So, in the case of probability theory there is simply no wedge to be driven between instrumental and normative rationality^1^.

This formulation also raises the question of how universal are the goals stated in the antecedent? In a conditional formulation the more universal an antecedent the less it needs to be stated. So, for example we would normally say *ripe apples fall*. We do not feel compelled to formulate this as *if gravity is in force ripe apples fall*. One could even use an appropriate modal, *ripe apples ought to fall*. Certainly one might be inclined to query whether this is a real or a good apple if it did not fall, which is perilously close to an evaluative judgment. Similarly, the more universal we regard the wish to avoid making bets one is bound to lose, the more inclined we would be to drop the conditional formulation and evaluate anyone not conforming to the rules of probability as irrational just as we may be inclined to evaluate the apple as inedible. If we encountered someone willing to make bets they were bound to lose, they would probably be institutionalized for their own safety. As with instrumental and normative rationality, the barrier between directive and evaluative rationality seems permeable. Moreover, the fundamental issue is of universality versus relativity. The theory is normatively rational if its justification is considered universal.

The inference to which Elqayam and Evans (2011) seem to take exception is the claim that as theoreticians we *should* accept the theory that makes the most rational sense of the participants’ behavior (Oaksford and Chater, 1996, 2007). As long as we are comparing the rules of normative theories, this will mean that the one that best describes participants’ behavior is the one that makes most rational sense of it. This thesis derives from the fact that in interpreting empirical data, i.e., our participants’ behavior, we are in exactly the same position as the *radical* interpreter in Davidson’s (1984, 2004) theory of ascribing intentional content. The difference is that as reasoning researchers we may have more than one normative theory in mind, whereas in radical interpretation one imputes one’s own norms to one’s interlocutor. However, the general principle remains the same: we are trying to make the best sense of what we have been told.

## INTERPRETATION, ARGUMENTATION, AND RATIONALITY

Davidson’s model of radical interpretation is an idealized account of how a cognitive agent can interpret another agent’s behavior and utterances to infer their beliefs and desires (Rescorla, 2013). The model is based on Bayesian decision theory, in which beliefs are graded and related to subjective probabilities and people’s desires are represented as utilities. Savage’s (1954) axioms show that when a person’s preferences meet certain requirements there are probabilities and utilities that guarantee that their preferences maximize expected utility. Consequently, an agent’s beliefs and desires can be inferred from their overt preferences. An important wrinkle is that the propositional content of beliefs are not pre-specified but are also inferred from an interlocutor’s preferences for the truth of sentences. Central to this account is the thesis that to ascribe another person with the appropriate beliefs and desires means we must assume they conform to our own standards of rationality. This is the principle of charity. As Davidson (2005; p. 319, cited in, Rescorla, 2013) puts it: “Charity is a matter of finding enough rationality in those we would understand to make sense of what they say and do, for unless we succeed in this, we cannot identify the contents of their words and thoughts.” Rationality is constitutive of having intentional states.

This is an idealized model but the central idea that we must attribute to others similar rational norms to ourselves in order to interpret them is intended as a more general claim about interpretation in the real world that involves attributing others with propositional attitudes like beliefs and desires. On Davidson’s view describing somebody’s behavior in terms of beliefs and desires is inseparable from normative evaluation.

Davidson’s (2004) emphasis on interpretive communicative processes proposes a particular research methodology which has been pursued recently in the context of human argumentation (Oaksford and Hahn, 2004, 2013; Hahn and Oaksford, 2007). Argumentation is a social phenomenon in which one or more people attempt to persuade another person or group of a particular, often controversial, position. It is a commonplace of argumentation theory that arguing is pointless unless there is broad agreement between the protagonists on what could count as a reasonable argument (Perelman and Olbrechts-Tyteca, 1969; Woods et al., 2004). Without this point of departure there is no point in engaging in an argumentative exchange. At least initially, we must apply the principle of charity^2^. Recently it has been argued that reasoning usually has an argumentative goal (Hahn and Oaksford, 2007; Mercier and Sperber, 2011). Consequently, it is in social argumentative contexts where people’s rational norms of reasoning would be expected to be most in evidence. It is a critical ability to be able to evaluate the arguments put forward by others to persuade you or your friends of particular positions.

Recent research in this area has adopted a third person argument evaluation methodology (Oaksford and Hahn, 2004, 2013; Hahn and Oaksford, 2007; Harris et al., 2012). Participants are explicitly asked to assess the degree to which one interlocutor, A, *should* be convinced by an argument put forward by another interlocutor, B. So, participants are explicitly asked for an evaluative judgment. They are also provided with information about A’s prior degree of belief in the conclusion. Hahn and Oaksford (2006, 2007), Oaksford and Hahn (2004, 2013) have provided normative Bayesian analyses of a variety of different forms of argumentation which make clear predictions for participants’ judgments. In this context, a normative Bayesian account provides excellent fits to the data. Moreover, this is true even when there are no parameters free to vary (Harris et al., 2012) because participants have been asked for their judgments of the relevant likelihoods from which predictions for their posteriors can be directly computed (see also, Fernbach and Erb, 2013). These results demonstrate that when participants are asked for an evaluative judgment of other peoples’ arguments they reveal behavior that is closely in accordance with the appropriate normative theory. This is not only because they have been asked directly to make an evaluative judgment. They are also explicitly provided with A’s prior degree of belief, which absolves them from the dilemma of considering whether *they* would believe the conclusion prior to hearing the argument. They are simply told that, for whatever reason, A believes it to a certain degree. In first person paradigms, participants are asked to assume or suppose that *they* believe the premises to be true or to a certain degree, when of course they may believe no such thing.

In summary, the psychology of reasoning will have to deal with evaluative normativity because much human behavior involves the explicit evaluation of others’ arguments, especially in politics, and in the law. Moreover, participants in experiments on argumentation make these evaluations naturally and their performance reveals direct sensitivity to appropriate rational norms.

## HOW MANY RATIONAL NORMS ARE THERE?

I conclude this paper by addressing two critical issues underlying Elqayam and Evans (2011) criticisms of evaluative normativity, (i) deciding between normative theories and (ii) the conviction that constructs like the principle of charity collapse into relativism. On Davidson’s (2004) ideal model there are no alternative normative frameworks. Basic logic, probability theory, and decision theory [see, Chater and Oaksford (2012) on the role of these theories in cognitive science] are fundamental rational norms and he broaches no other possibilities. This raises the question, of how many rational norms are there actually to choose between? A prima facie argument can be made that that there are not as many as one might think. Elqayam and Evans (2011) suggest that the new Bayesian paradigm is an alternative norm account. I argue that since probability theory presupposes standard logic they are not really in competition. A derived theorem of the Kolmogorov axioms is *logical consequence*, i.e., *if X logically entails Y, then Pr*(*Y*) ≥ *Pr*(*X*), which “ensures that probabilistic reasoning respects deductive logic” (Joyce, 2004, p. 135). The question is not whether one norm supplants another but whether beliefs are graded. Once we opt for graded beliefs, then we need to know how they are updated in inference when new information comes in. This can be achieved by Bayesian conditionalization rather than modus ponens (Oaksford, in press; Oaksford and Chater, 2007, 2013), although this is not necessary because probabilistic premises will *deductively* entail a probability interval for the conclusions of an argument (Pfeifer and Kleiter, 2010). Consequently, I suggest that the move to Bayesian probability is not a move to an alternative norm rather than a move to a finer grained analysis of beliefs which is not just binary true or false.

Thus, when comparing logic and probability, we are not choosing between competing norms. Davidson would argue, and common sense seems to dictate, that if the more nuanced view provides a rational understanding of more of the data it is the preferred theory. When the issue of competing norms is taken out of the equation this is simply the question of which theory provides the best description of the data. What happens if there are genuinely competing normative theories that are equally descriptively adequate?

For example, in decision theory an explicit competitor to classical Bayesian probability theory has been provided by quantum probability (Pothos and Busemeyer, 2013). This would appear to be much closer to the competing norms case that Elqayam and Evans (2011) envisage. Quantum probability stands to quantum logic – in which the law of the excluded middle is not valid – as Bayesian probability stands to standard logic (Oaksford, 2013). Moreover, across a variety of tasks, Pothos and Busemeyer (2013) argue that quantum probability is more descriptively adequate than Bayesian probability theory. Recall that the formulation for directive normativity is conditional, with the relevant justification for the normative theory in the antecedent. For Bayesian probability theory we have, *if an agent wishes to avoid making bets they cannot win, then they should conform to the laws of probability theory*. For quantum probability, however, there does not appear to be a relevant justificatory antecedent. There would appear to be no Dutch book theorem showing that failure to conform to the laws of quantum probability would lead anyone to make bets they could not win^3^. Moreover, conformity to the laws of quantum probability may well lead to a Dutch book being made against you. For example, it has been shown that committing the conjunction fallacy (Tversky and Kahneman, 1983) can allow a Dutch book to be made against you (Gilio and Over, 2012; Hahn 2014) and quantum probability apparently *predicts* the conjunction fallacy (Pothos and Busemeyer, 2013). Consequently, however descriptively adequate with respect to the data quantum probability appears to be, it cannot explain how behavior succeeds in the real macroscopic world which we inhabit. Even if we can make sense of laying bets on the outcomes of quantum events, there would still need to be an independent argument that there are similar events about which we could gamble at the macroscopic level (Oaksford, 2013).

Elqayam and Evans (2011) argue against the principle of charity solely on the observation that norms are relative to particular cultural and historical contexts. However, they do not discuss Davidson’s view of rationality as a constitutive norm (Rescorla, 2013). On Davidson’s view conformity to these norms is constitutive of having intentional states and is not relative to any particular cultural or historical context. As there are no human beings to whom we would not attribute beliefs this suggests that our norms are also universal. The Dutch book theorems certainly have this character. Gambling is a universal human activity, engaged in by all cultures and in all historical contexts. Moreover, it seems inconceivable that anyone would fail to accede to the rationale for the Dutch book theorems, what normal human being would wish to make bets they are bound to lose? In the first section, I argued that the permeability between directive and evaluative rationality depends on the universality of the justification for a normative system. So we have good grounds to view probability theory as a universal evaluative norm.

## CONCLUSION

In conclusion, in the psychology of reasoning, interpreting experimental results, just as in interpreting another’s utterances, requires making the best rational sense of the observed behavior. People evaluate each other’s arguments in politics and in the law and in appropriate argumentative contexts their judgments conform to the rational norms of probability theory. The current Bayesian turn in the psychology of reasoning addresses the question of whether beliefs are graded and is not an alternative norm to standard logic. From Davidson’s perspective, the universal attribution of beliefs to others has the corollary that our rational norms are likely to be similarly universal. Elqayam and Evans (2011) provide no grounds to question this perspective. However, there are many exceptions, data that does not conform to these norms (e.g., Tversky and Kahneman, 1983; but see, Crupi et al., 2008), cases of irrationality due to illness or injury, cases where sacred values are opposed to utility maximization (Atran and Axelrod, 2008), and other paradoxes of maximizing expected utility (Burns and Wieth, 2004; but see Turner and Quilter, 2014). However, there are responses to these exceptions as some of the citations indicate. In sum, the union of evaluative normativity and descriptive psychology, implicit in Davidson (Rescorla, 2013), is continuing to yield important results and this should be regarded as a good thing.

## Conflict of Interest Statement

The author declares that the research was conducted in the absence of any commercial or financial relationships that could be construed as a potential conflict of interest. The Associate Editor declares that while the author Mike Oaksford as well as the reviewer Ulrike Hahn are currently affiliated with the same department (Department of Psychological Sciences, Birkbeck College, University of London), there has been no conflict of interest during the review and handling of this manuscript.

## Footnotes

1 We note also that the justification for selecting data in accordance with Oaksford and Chater’s (1994) information gain model is again instrumental. So following its dictates will mean that this strategy minimizes the length of the sequential sample needed for the posteriors to converge on the true hypothesis (Fedorov, 1972). This is an instrumental justification: *if* people want to get to the truth in the most economical way they will select data in accordance with the theory.

2 After an initial exchange, we may discover that we are not in a critical discussion, i.e., a rational exchange of arguments intended to persuade, but rather are in a quarrel, where rationality goes out the window.

3 Although in physics, there are arguments that a Bayesian approach, i.e., probability as a measure of ignorance, might make sense of quantum probability as a theory of rational betting in quantum gambles (Pitowsky, 2003). One then has to ask whether there is any analog of a quantum gamble at the macroscopic level that any human being would be concerned to win.
